# Metastasis pattern and prognosis in children with neuroblastoma

**DOI:** 10.1186/s12957-023-03011-y

**Published:** 2023-04-12

**Authors:** Shan Liu, Weimin Yin, Yaobin Lin, Sihan Huang, Shufang Xue, Gaoyuan Sun, Chengyi Wang

**Affiliations:** 1 Department of Hematology–Oncology, Fujian Children’s Hospital, Fujian Medical University, Fuzhou, Fujian China; 2 College of Clinical Medicine for Obstetrics and Gynecology and Pediatrics, Fujian Medical University, Fuzhou, Fujian China; 3 Clinical Oncology School of Fujian Medical University, Fujian Cancer Hospital, Fuzhou, Fujian China

**Keywords:** Neuroblastoma, Distant metastases, Overall survival, Cancer-specific survival, SEER

## Abstract

**Background:**

We aimed to investigate the different metastases and prognoses of neuroblastoma (NB) and determine the risk factors of metastasis.

**Method:**

Data of 1224 patients with NB were obtained from the Surveillance, Epidemiology and End Results database (2010–2018). Pearson’s chi-square test, Kaplan–Meier analysis, multivariable logistic regression and Cox regression analysis were used to determine the factors associated with prognosis.

**Results:**

The overall incidence of NB was an age-adjusted rate of 8.2 patients per 1,000,000 children. In total, 1224 patients were included in our study, with 599 patients (48.9%) exhibiting distant metastases. Compared to patients with non-metastatic NB, a greater proportion of patients with metastatic NB were under 1 year, male, had an adrenal primary site, unilateral tumour, a tumour size > 10 cm, neuroblastoma-not otherwise specified (NB-NOS), second malignant neoplasms and were more likely to choose radiotherapy and chemotherapy. Multivariate Cox regression showed that metastasis was an independent risk factor for overall survival (OS) and cancer-specific survival (CSS). The survival rate of non-metastatic patients with NB was better than those with metastasis (OS: hazard ratio (HR): 0.248, *P* < 0.001; CSS: HR: 0.267, *P* < 0.001). The bone and liver were the two most common isolated metastatic sites in NB. However, no statistical difference was observed in OS and CSS between the only bone metastasis group, only liver metastasis group and bone metastasis combined with liver metastasis group (all *P* > 0.05). Additionally, age at diagnosis > 1 year (odds ratio (OR): 3.295, *P* < 0 .001), grades III–IV (OR: 26.228, *P* < 0 .001) and 5–10 cm tumours (OR: 1.781, *P* < 0 .001) increased the risk of bone metastasis of NB. Moreover, no surgical treatment (OR: 2.441, *P* < 0 .001) increased the risk of liver metastasis of NB.

**Conclusion:**

Metastatic NB has unique clinicopathological features, with the bone and liver as the most common single metastatic sites of NB. Therefore, more aggressive treatment is recommended for high-risk children with NB displaying distant metastases.

## Background

Neuroblastoma (NB) is a highly metastatic malignant tumour, with 70% of patients exhibiting metastasis at the time of diagnosis [1], along with poor prognosis and resistance to conventional chemotherapy. The most common sites of distant metastasis are reported to be the bone marrow and bone, followed by the liver and regional lymph nodes. The 5-year survival rate of low- and medium-risk children has been estimated to reach 75–98% but that of high-risk children is less than 50% [2, 3]. Relapse or progression at the metastatic site is a major cause of NB-related mortality [4].

Currently, internationally recognised treatment-based risk stratification of NB include age, histology, grade, MYCN, 11q aberration and DNA ploidy [5, 6]. The 5-year survival rate of patients with metastatic NB has increased from 20 to > 50% [7], largely owing to the combination of high-dose chemotherapy and autologous stem cell transplantation and differentiation agents and immunotherapy with anti-GD2 monoclonal antibody [8]. However, only a few large-scale clinical studies on the metastasis pattern of NB in children have been reported.

Therefore, this study aims to use the Surveillance, Epidemiology, and End Results (SEER) database to analyse the metastatic pattern of NB, screen for metastasis-related risk factors and determine the clinicopathological features and independent prognostic factors of metastatic patients.

## Materials and methods

### Patients and ethics

We used the SEER database and SEER-stat software (SEER*Stat 8.3.9) to identify and collect the data of patients aged ≤ 18 years with a confirmed NB diagnosis according to the International Classification of Diseases for Oncology (ICD-O-3) between 2010 and 2018. Data on the following variables were extracted: age at diagnosis, gender, race, year of diagnosis, primary site, tumour grade, laterality, tumour size, histology, chemotherapy, surgery, radiotherapy, second malignant neoplasms (SMN), metastasis, survival time and cause of death. SMN was defined as the appearance of other malignant tumours at least 2 months after NB diagnosis.

Tumour grades were defined as follows: (1) grade I, also called well-differentiated; (2) grade II, moderately differentiated; (3) grade III, poorly differentiated; and (4) grade IV, undifferentiated or anaplastic.

The metastasis of distant organs is defined in SEER as the state of metastasis of distant organs at the time of the first diagnosis of cancer. Among the distant organs, bone metastasis did not include bone marrow metastasis.

**Fig. 1:**
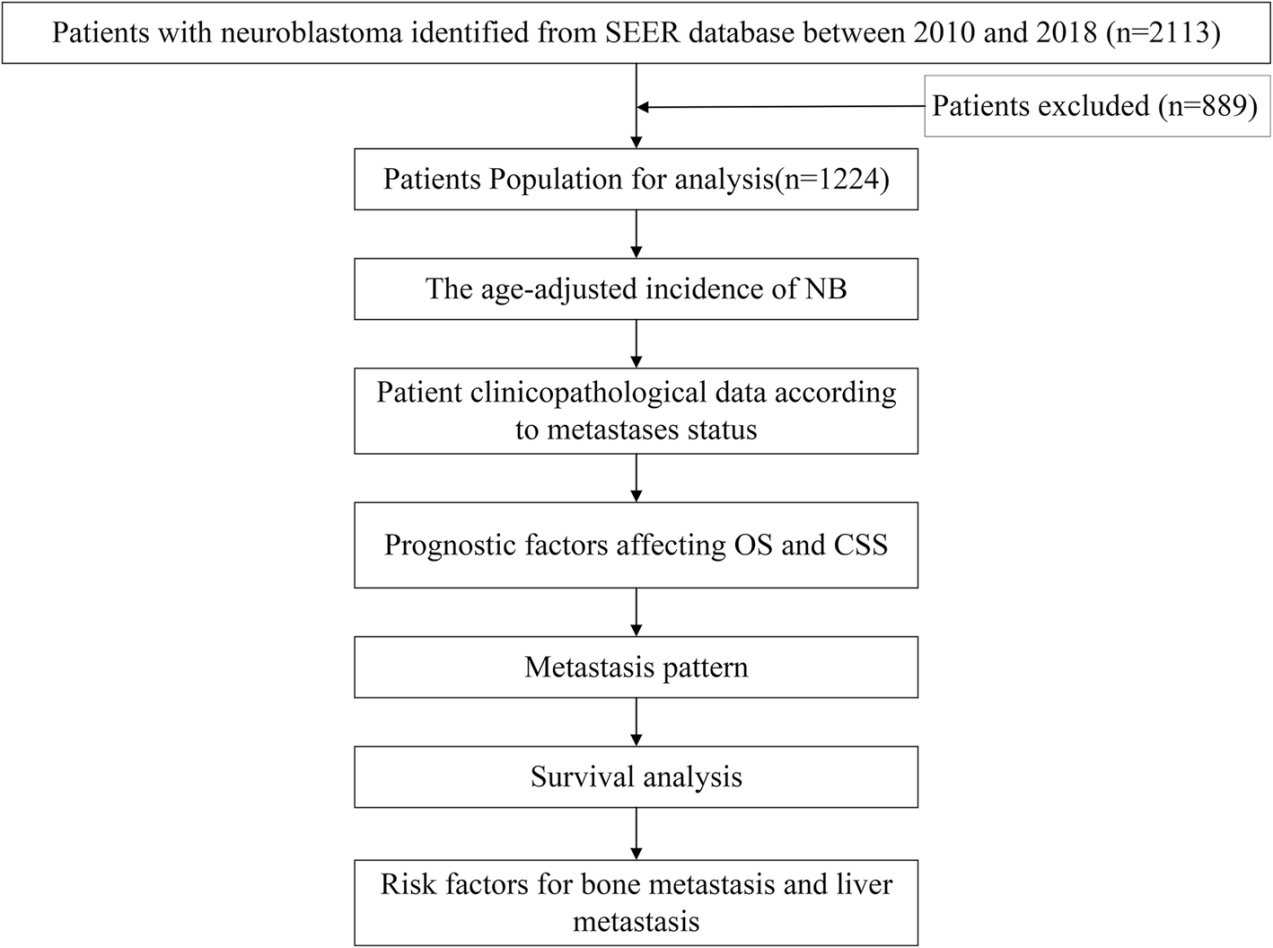
Flow chart of the overall study design

The inclusion criteria were as follows: (1) patients with ICD-O-3 Hist/behave = neuroblastoma (ICD-O-3 = 9500) or ganglioneuroblastoma (ICD-O-3 = 9490) and (2) patients with NB as the primary tumour. The exclusion criteria were as follows: (1) lack of follow-up data; (2) diagnosis not confirmed by pathological examinations; and (3) age > 18 years. The research flow is presented in Fig. 1.

All data in the SEER database are anonymised; therefore, ethical approval was not required for this study.

### Statistical analysis

The age-standardised incidence of NB based on the 2000 US Standard Population was identified from the cancer registries of the SEER database. The annual percentage change (APC) of incidence was calculated using the weighted least squares method. Moreover, as the cause of death of seven children was unknown, CSS was excluded from the analysis. Kaplan–Meier was used for survival analysis and between-group differences were assessed using Pearson’s chi-square test and Fisher’s exact probability method. Univariate Cox analysis was performed to identify factors (*P* < 0.05) for inclusion in the multivariate Cox regression models. Furthermore, logistic regression was used to estimate the odds ratio (OR) of metastasis in patients with NB.

Data analyses were performed using SPSS 26.0 for Windows (Armonk, NY: IBM Corp) and R software (version 3.6.3), namely the ‘survival’ and ‘ggplot2’ packages. *P* < 0.05 was considered statistically significant.

## Results

### The age-adjusted incidence of NB

**Fig. 2:**
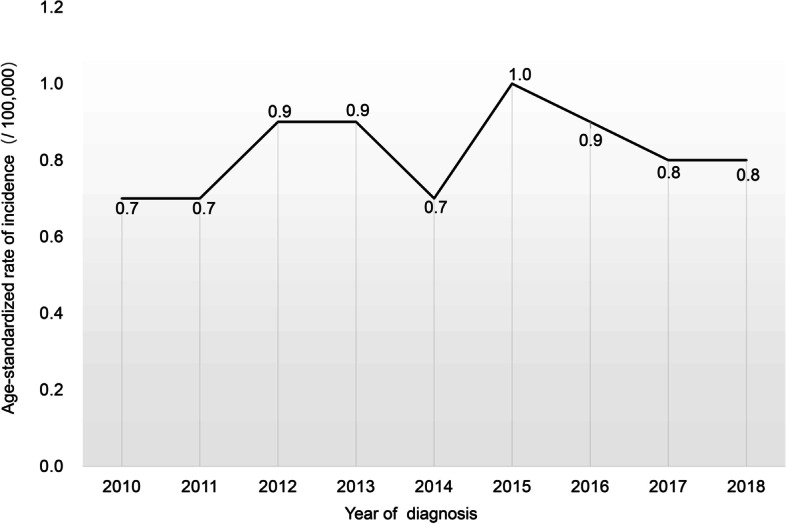
The age-adjusted incidence of NB in the USA (2010–2018). Abbreviations: NB, neuroblastoma

A total of 1224 patients were included in our study. The median follow-up time was 42.5 months (inter-quartile range (IQR) 15.0–67.0 months). The overall age-adjusted invasive NB incidence rate for SEER from 2010 to 2018 was 8.2 per 1,000,000 children annually. Notably, the annual incidence rate showed no downtrend decline annually. Furthermore, the APC was 0.1% [95% confidence interval (CI), − 0.2 to 0.4%; *P* > 0.05] (Fig. 2).

### Patient clinicopathological data according to metastases status

A total of 599 (48.9%) patients displayed distant metastases. Moreover, 1003 (81.9%) tumours were NB-not otherwise specified (NOS) and 221 (18.1%) were ganglioneuroblastoma. Table 1 shows the baseline characteristics of the patients. The two groups had significant differences in age, gender, primary site, laterality, tumour size, histology and SMN (all *P* ≤ 0.05). Furthermore, the metastatic group underwent more radiation therapy and chemotherapy and had lesser surgical interventions than the non-metastatic group. However, both groups were identical in race and tumour grade.

**Table 1:** Characteristics of patients with metastatic neuroblastoma

**Variable**	***N* (%)**	**NB with metastasis,** ***N* (%)**	**NB without** **metastasis,** ***N* (%)**	***P***
Total	1224 (100.0)	599 (100.0)	625 (100.0)	
**Age (years)**				< 0.001
≤ 1	611 (49.9)	257 (42.9)	354 (56.6)	
> 1	613 (50.1)	342 (57.1)	271 (43.4)	
**Gender**				0.024
Male	632 (51.6)	329 (54.9)	303 (48.5)	
Female	592 (48.4)	270 (45.1)	322 (51.5)	
**Race**				0.338
White	884 (72.2)	432 (72.1)	452 (72.3)	
Black	194 (15.8)	102 (17.0)	92 (14.7)	
Others/unknown	146 (11.9)	65 (10.9)	81 (13.0)	
**Primary site**				< 0.001
Adrenal	577 (47.1)	387 (64.6)	190 (30.4)	
Soft tissue	304 (24.8)	213 (35.6)	91 (14.6)	
Retroperitoneum	124 (10.1)	53 (8.8)	71 (11.4)	
Others	219 (17.9)	68 (11.4)	151 (24.2)	
**Tumour grade**				0.240
Grades I–II	15 (1.2)	4 (0.7)	11 (1.8)	
Grades III–IV	608 (49.7)	316 (52.8)	292 (46.7)	
Unknown	611 (49.9)	279 (46.6)	332 (53.1)	
**Laterality**				< 0.001
Unilateral	709 (57.9)	428 (71.5)	281 (45.0)	
Bilateral	27 (2.2)	15 (2.5)	12 (1.9)	
Unknown	488 (39.9)	156 (26.0)	332 (53.1)	
**Tumour size (cm)**				< 0.001
5	221 (18.1)	75 (12.5)	146 (23.4)	
5–10	318 (26.0)	157 (26.2)	161 (25.8)	
10	141 (11.5)	88 (14.7)	53 (8.5)	
Unknown	544 (44.4)	279 (46.6)	265 (42.4)	
**Histology**				< 0.001
Neuroblastoma-NOS	1003 (81.9)	563 (94.0)	440 (70.4)	
Ganglioneuroblastoma	221 (18.1)	36 (6.0)	185 (29.6)	
**Chemotherapy**				< 0.001
Yes	829 (67.7)	565 (94.3)	264 (42.2)	
No	395 (32.3)	34 (5.7)	361 (57.8)	
**Surgery**				0.001
Yes	939 (76.7)	436 (72.8)	503 (80.5)	
No	285 (23.3)	163 (27.2)	122 (19.5)	
**Radiotherapy**				< 0.001
Yes	321 (26.2)	259 (43.2)	62 (9.9)	
No	903 (73.8)	340 (56.8)	563 (90.1)	
**SMN**				< 0.001
Yes	25 (2.0)	21 (3.5)	4 (0.6)	
No	1199 (98.0)	578 (96.5)	621 (99.4)	

*Abbreviations: SMN* second malignant neoplasms, *NB* neuroblastoma, *NOS* not otherwise specified, *N* number

### Prognostic factors affecting overall survival (OS) and cancer-specific survival (CSS)

Univariate analysis showed that age, primary site, laterality, tumour size, histology, chemotherapy, radiotherapy and metastasis were the risk factors for OS and CSS (all *P* < 0.05) (Table 2). Additionally, multivariate Cox regression analyses revealed that tumour size > 10 cm and distant metastasis correlated with poor OS (Table 3). Furthermore, tumour size ≥ 5 cm, lack of chemotherapy and distant metastasis correlated with poor CSS. The survival of patients with no metastases was better than those with metastases (OS: hazard ratio (HR): 0.248, *P* < 0.001; CSS: HR: 0.267, *P* < 0.001).

**Table 2:** Univariate Cox proportional hazard regression model of OS and CSS

Variable	OS univariable analysis		CSS univariable analysis	
	**Hazard ratio (95% CI)**	***P* value**	**Hazard ratio (95% CI)**	***P*** **value**
**Age (years)**				
≤ 1	Reference	–	Reference	–
> 1	1.683 (1.252–2.262)	0.001	1.704 (1.239–2.343)	0.001
**Gender**				
Male	Reference	–	–	–
Female	0.866 (0.649–1.157)	0.330	1.010 (0.742–1.377)	0.947
**Race**		0.644		0.952
White	Reference	–	Reference	–
Black	1.184 (0.815–1.721)	0.375	0.977 (0.639–1.495)	0.915
Other/unknown	1.111 (0.707–1.747)	0.647	1.071 (0.659–0.1.739)	0.783
**Primary site**		< 0.001		< 0.001
Adrenal	Reference	–	Reference	–
Soft tissue	0.307 (0.197–0.480)	< 0.001	0.273 (0.166–0.449)	< 0.001
Retroperitoneum	0.519 (0.299–0.902)	0.020	0.506 (0.279–0.919)	0.025
Others	0.529 (0.346–0.808)	0.003	0.532 (0.339–0.835)	0.006
**Tumour grade**		0.353		0.264
Grades I–II	Reference	–	Reference	–
Grades III–IV	2.890 (0.404–20.702)	0.291	2.572 (0.359–28.464)	0.347
Unknown	2.471 (0.344–17.775)	0.369	2.055 (0.285–14.819)	0.475
**Laterality**		< 0.001		< 0.001
Unilateral	Reference	–	Reference	
Bilateral	0.970 (0.429–2.195)	0.942	0.928 (0.380–2.269)	0.870
Unknown	0.324 (0.224–0.468)	< 0.001	0.319 (0.241–0.474)	< 0.001
**Tumour size (cm)**		< 0.001		< 0.001
< 5	Reference	–	Reference	–
5–10	2.472 (1.463–4.178)	0.001	2.874 (1.600–5.162)	< 0.001
> 10	4.124 (2.374–7.164)	< 0.001	5.063 (2.754–9.3060)	< 0.001
Unknown	2.579 (1.524–4.365)	< 0.001	2.679 (1.478–4.855)	0.001
**Histology**		< 0.001		0.001
Neuroblastoma-NOS	Reference	–	Reference	–
Ganglioneuroblastoma	0.427 (0.256–0.712)	0.001	0.398 (0.226–0.701)	0.001
**Chemotherapy**		< 0.001		< 0.001
Yes	Reference	–	Reference	–
No	0.204 (0.124–0.336)	< 0.001	0.149 (0.081–0.274)	< 0.001
**Surgery**				
Yes	Reference	–	Reference	–
No	1.178 (0.844–1.644)	0.336	1.071 (0.742–1.547)	0.713
**Radiotherapy**				
Yes	Reference	–	Reference	–
No	0.417 (0.313–0.557)	< 0.001	0.374 (0.274–0.509)	< 0.001
**SMN**				
Yes	Reference	–	Reference	–
No	0.615 (0.289–1.308)	0.207	0.948 (0.351–2.557)	0.915
**Metastasis**				
Yes	Reference	–	Reference	–
No	0.153 (0.102–0.229)	< 0.001	0.144 (0.092–0.223)	< 0.001

*Abbreviations: CI* confidence interval, *OS* overall survival, *CSS* cancer-specific survival, *SMN* second malignant neoplasms, *NOS* not otherwise specified

**Table 3:** Multivariate Cox proportional hazard regression model of OS and CSS

Variable	OS multivariate analysis		CSS multivariate analysis	
	**Hazard ratio (95% CI)**	***P* value**	**Hazard ratio (95% CI)**	***P* value**
**Age (year)**				
≤ 1	Reference	–	Reference	–
> 1	1.286 (0.926–1.787)	0.133	1.255 (0.882–1.786)	0.207
**Primary site**		0.282		0.221
Adrenal	Reference	–	Reference	–
Soft tissue	0.989 (0.561–1.744)	0.971	0.847 (0.450–1.597)	0.608
Retroperitoneum	1.342 (0.681–2.642)	0.395	1.232 (0.587–2.584)	0.582
Others	1.520 (0.940–2.457)	0.088	1.539 (0.917–2.582)	0.102
**Laterality**		0.003		0.017
Unilateral	Reference	–	Reference	–
Bilateral	1.088 (0.470–2.520)	0.843	1.123 (0.448–2.818)	0.804
Unknown	0.407 (0.241–0.689)	0.001	0.440 (0.247–0.783)	0.005
**Tumour size (cm)**		0.013		0.006
< 5	Reference	–	Reference	–
5–10	1.948 (1.145–3.313)	0.014	2.200 (1.217–3.979)	0.009
> 10	2.584 (1.465–4.560)	0.001	3.066 (1.642–5.725)	< 0.001
Unknown	1.999 (01.172–3.412)	0.011	2.044 (1.118–3.735)	0.020
**Histology**				
Neuroblastoma-NOS	Reference	–	Reference	–
Ganglioneuroblastoma	0.904 (0.515–1.589)	0.727	0.943 (0.508–1.747)	0.851
**Chemotherapy**				–
Yes	Reference	–	Reference	–
No	0.623 (0.343–1.133)	0.121	0.451 (0.221–0.918)	0.028
**Radiotherapy**				
Yes	Reference	–	Reference	–
No	0.998 (0.722–1.380)	0.990	0.941 (0.666–1.330)	0.731
**Metastasis**				
Yes	Reference	–	Reference	–
No	0.250 (0.155–0.403)	< 0.001	0.268 (0.161–0.447)	< 0.001

*Abbreviations: CI*, confidence interval; *OS*, overall survival; *CSS*, cancer-specific survival; *NOS*, not otherwise specified

### Metastasis pattern

As shown in Table 4, NB has 15 possible metastatic forms, including 4 single metastases and 11 combinations of metastases. Among patients with single-organ metastasis, we found that the bone was the most common site of metastasis (42.6%), followed by the liver (10.9%), lung (1.3%) and brain (0.2%). For multiple site metastases, the combination of bone and liver metastases was estimated to be 9.5%, accounting for the majority of all cases. Additionally, 329 (54.9%), 107 (17.9%), 22 (3.7%) and 2 (0.3%) patients had one, two, three and four sites of metastasis of NB, respectively.

**Table 4:** Organ metastasis patterns in patients with neuroblastoma

Variable	*N* (%)	≤ 1 year, *N* (%)	> 1 year, *N* (%)	*P*
Total	599 (100%)	257 (%)	342 (%)	–
Bone metastasis only	255 (42.6)	71 (27.6)	184 (53.8)	< 0.001
Brain metastasis only	1 (0.2)	1 (0.4)	0 (0.0)	0.248
Liver metastasis only	65 (10.9)	58 (22.6)	7 (2.0%)	< 0.001
Lung metastasis only	8 (1.3)	3 (1.2)	5 (1.5)	0.756
Bone and brain	29 (4.8)	9 (3.5)	20 (5.8)	0.186
Bone and liver	57 (9.5)	34 (13.2)	23 (6.7)	0.007
Bone and lung	17 (2.8)	4 (1.6)	13 (3.8)	0.102
Brain and liver	0 (0.0)	0 (0.0)	0 (0.0)	–
Brain and lung	0 (0.0)	0 (0.0)	0 (0.0)	–
Liver and lung	4 (0.7)	4 (1.6)	0 (0.0)	0.021
Bone, brain and liver	5 (0.8)	2 (0.8)	3 (0.9)	0.895
Bone, brain and lung	2 (0.3)	1 (0.4)	1 (0.3)	0.839
Bone, liver and lung	15 (2.5)	9 (3.5)	6 (1.8)	0.175
Brain, liver and lung	0 (0.0)	0 (0.0)	0 (0.0)	–
Bone, brain, liver and lung	2 (0.3)	2 (0.8)	0 (0.0)	0.102
One site metastasis	329 (54.9)	133 (51.8)	196 (57.3)	0.176
Two-site metastases	107 (17.9)	51 (19.8)	56 (16.4)	0.272
Three-site metastases	22 (3.7)	12 (4.7)	10 (2.9)	0.261
Four-site metastases	2 (0.3)	2 (0.8)	0 (0.0)	0.102

*Abbreviations: N* number

Furthermore, we compared the differences in metastasis pattern between the ≤ 1-year group and > 1-year group, wherein the > 1-year group had a higher incidence of bone metastasis and a lower incidence of liver metastasis than the ≤ 1-year group (bone metastasis only: 27.6% *vs* 53.8%, *P* < 0.001; liver metastasis only: 22.6% *vs* 2.0%, *P* < 0.001). In terms of multiple organ metastasis, the incidence of bone + liver and liver + lung in the ≤ 1-year group was higher than that in > 1 year group (bone + liver: 13.2% *vs* 6.7%, *P* = 0.007; liver + lung: 1.6% *vs* 0.0%, *P* = 0.021).

### Survival analysis

**Fig. 3:**
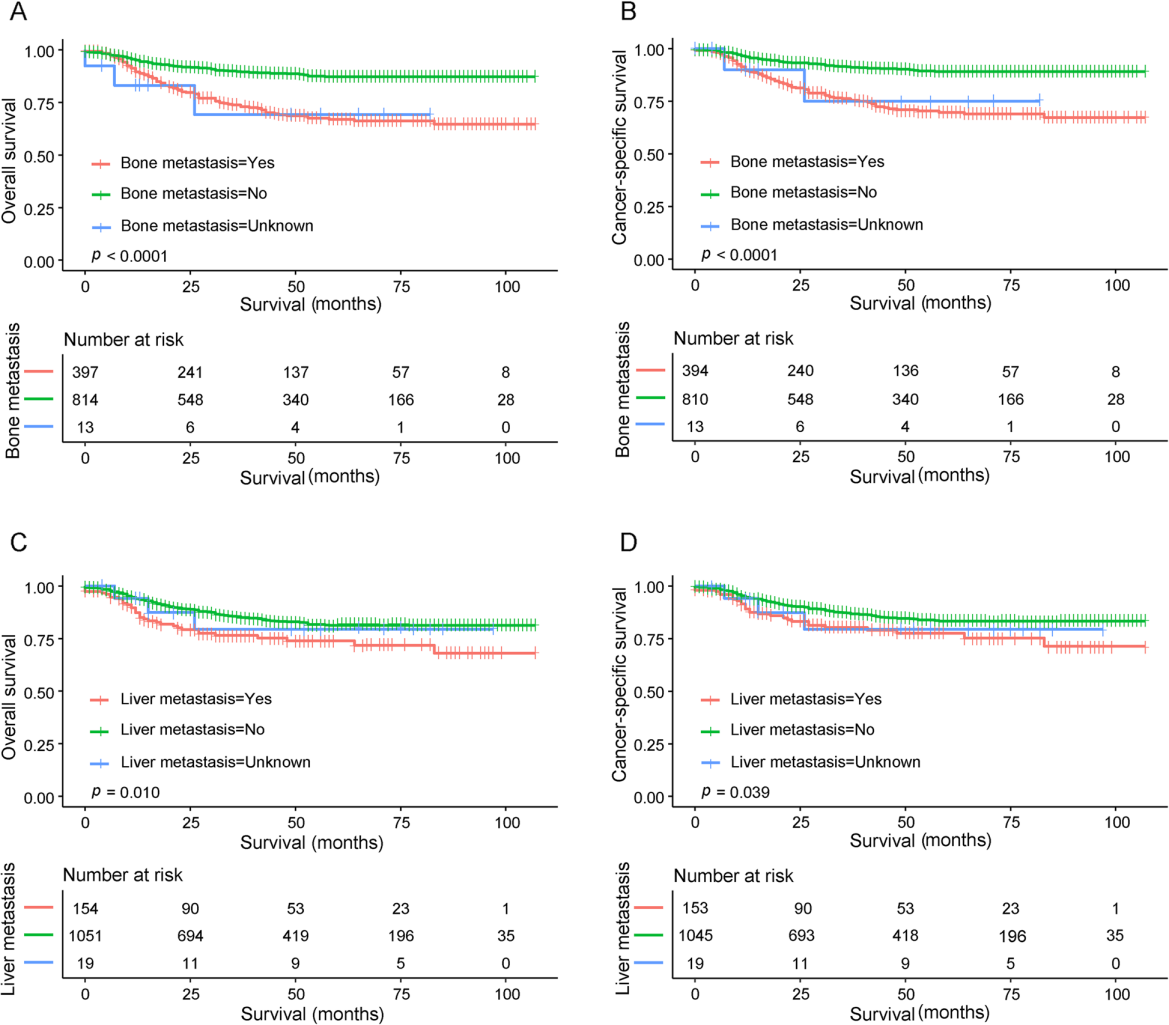
Kaplan-Meier curves for OS and CSS in NB patients with a single site of metastasis. **A**, **B** OS and CSS between patients with and without bone metastasis. **C**, **D** OS and CSS between patients with and without liver metastasis. Abbreviations: OS, overall survival; CSS, cancer-specific survival; NB, neuroblastoma

**Fig. 4:**
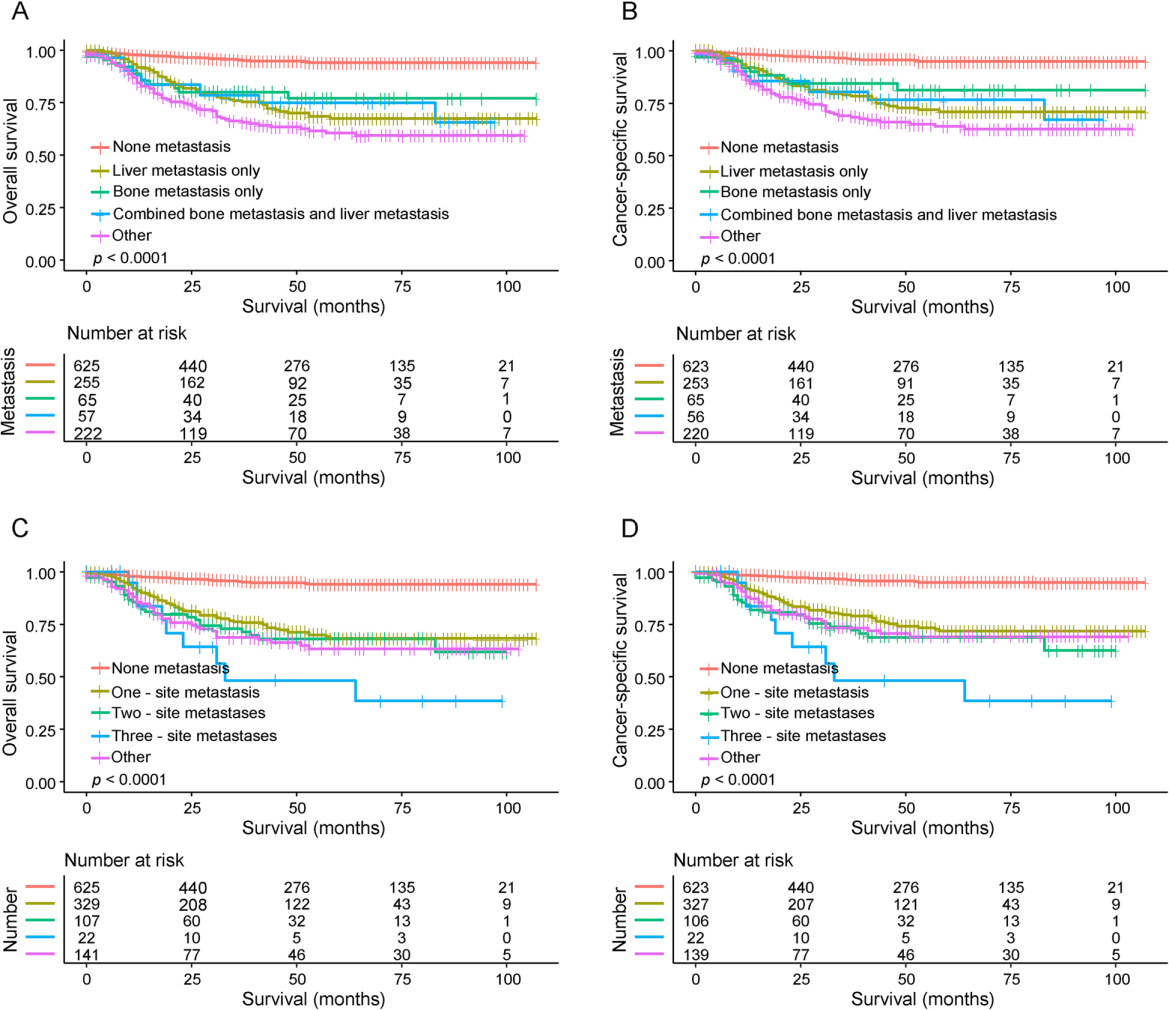
Kaplan–Meier curves for OS and CSS of patients with NB at different metastasis sites. **A**, **B** The survival difference among the different metastasis sites in patients with NB. **C**, **D** The survival difference among the different numbers of metastasis sites in patients with NB. Abbreviations: OS, overall survival; CSS, cancer-specific survival; NB, neuroblastoma

Kaplan–Meier analysis revealed that the patients without bone metastasis had a better 5-year OS and CSS than those with bone metastasis (OS: 87.2% vs 66.9%, *P* < 0.001; CSS: 89.1% vs 69.7%, *P* < 0.001, Fig. 3A, B). Similar results were also seen in patients without and with liver metastasis (OS: 81.3% vs 74%, *P* = 0.010; CSS: 83.4% vs 77.6%, *P* = 0.038, Fig. 3C, D). As bone and liver metastases were most commonly observed in NB, survival differences were observed between the different combinations in this study. As shown in Fig. 4A, B, the OS and CSS of the metastasis group were worse than the without metastasis group. Furthermore, no statistical difference was observed between the only bone metastasis group, only liver metastasis group and the combined bone and liver metastases group (all* P* > 0.05). As shown in Fig. 4C, D, survival rates decreased as the number of metastatic sites increased in patients with NB.

### Risk factors for bone metastasis and liver metastasis

**Fig. 5:**
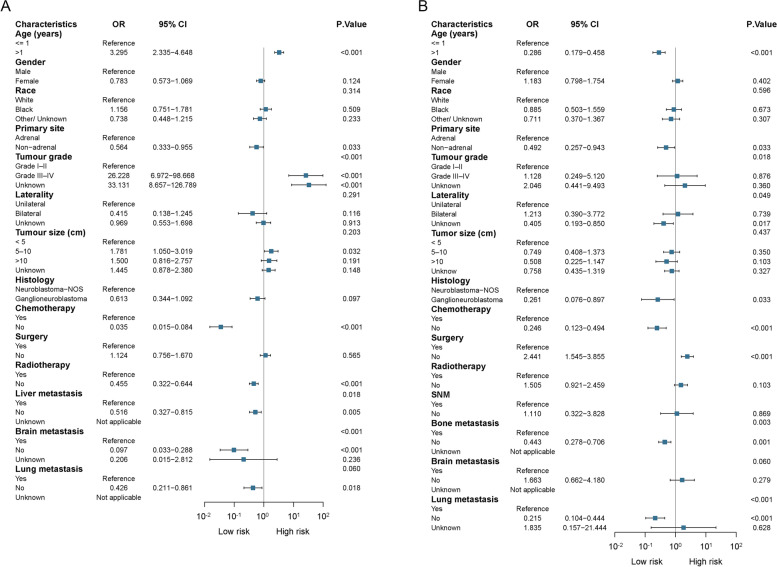
The risk factors for the different primary sites in the developing metastatic disease. **A** Bone metastasis. **B** Liver metastasis. Abbreviations: SMN, second malignant neoplasms; CI, confidence interval; OR, odds ratio; NOS, not otherwise specified

Multivariate logistic regression analysis revealed that age at diagnosis > 1 year (OR: 3.295, 95% CI: 2.335–4.648; *P* < 0 0.001), tumour grades III–IV (OR: 26.228, 95% CI: 6.972–98.668, *P* < 0 0.001) and tumour size 5–10 cm (OR: 1.781, 95% CI: 1.050–3.019, *P* < 0 0.001) increased bone metastasis risk in NB. However, a non-adrenal primary site and lack of chemotherapy or radiotherapy without liver, brain or lung metastasis decreased the risk of bone metastasis (Fig. 5A).

When screening the risk factors of hepatoma with liver metastasis, we observed that a lack of surgery (OR: 2.441, 95% CI: 1.545–3.855, *P* < 0 0.001) increased liver metastasis risk in NB; however, diagnosis > 1 year, non-adrenal primary site, ganglioneuroblastoma, lack of chemotherapy and lack of bone or lung metastasis decreased the risk of liver metastasis (Fig. 5B).

## Discussion

NB is a biologically and clinically heterogeneous group of tumours. It is also the most common paediatric extracranial solid tumour and is responsible for up to 15% of all paediatric oncologic deaths [9]. According to our results, the overall age-adjusted incidence rate of NB was 8.2 per 1,000,000 children annually, and the annual incidence rate showed no significant decrease, which could be highly correlated to the lack of specific clinical manifestations and detection methods in the early stage of the disease. A recent study revealed that nearly 80% of patients with metastatic NB show an ineffective response to current treatment methods [10]. This study compared the clinical features and prognosis of metastatic and non-metastatic NB and screened out high-risk factors for metastasis, thereby aiding early diagnosis. In addition to conventional surgery, chemotherapy and radiotherapy, for high-risk children with distant metastasis, combined tandem autologous transplantation or targeted immunotherapy can be considered.

In this study, 48.9% of patients with NB displayed distant metastasis at the time of diagnosis, which is consistent with previous reports [11]. Compared to patients with non-metastatic NB, a higher proportion of patients with metastatic NB with age > 1 year, male, the presence of an adrenal primary site, unilateral tumours, a tumour size > 10 cm, NB-NOS and SMN features were observed. Consistent with the previous report, our results also showed that primary adrenal NB is prone to metastasis [12, 13]. These differences in biological behaviour could be attributed to the fact that (1) primary adrenal neuroblastoma is more likely to carry structural DNA distortion, including MYCN amplification [13], and (2) the tumour microenvironment is different [12–14]. Accordingly, patients with metastatic NB often lose the opportunity of surgery and are more likely to choose radiotherapy and chemotherapy. However, we compared the OS and CSS of the non-metastasis group, the bone metastasis-only group, the liver metastasis-only group and the combined bone and liver metastasis group and found that the survival prognosis of children with distant metastasis was poor, indicating that conventional therapies such as chemotherapy, surgery and radiotherapy have limited efficacy in the treatment of metastatic NB.

Currently, new therapies for NB mainly include immunotherapy, targeted therapy, tandem-autologous transplantation or a combination of multiple methods. In immunotherapy, the combination of GD2 monoclonal antibody (mAb), chemotherapy regimen and autologous NK cells or the combination of anti-GD2 antibody and other antibodies or CART, such as CD47 mAb, have been reported to improve the tumour immunosuppressive microenvironment and enhance the therapeutic effect [15, 16]. Cheung et al. reported that the anti-GD2 antibody has good efficacy in high-risk NB, with an OS of 71% ± 7% at 5 years [17]. Regarding targeted therapy, currently, the majority of research is focused on the ALK, RAS-MAPK and N-Myc-Myc pathways. Mueller et al. reported that 22 (41.5%) of the 53 children treated with chemotherapy combined with anti-GD2 antibody had a shrunken tumour size or underwent complete remission and the 1-year OS rate reached up to 90% [18]. Compared with the previous single transplantation, tandem autologous stem cell transplant showed a better therapeutic effect in high-risk NB. Park et al. reported a clinical trial of 652 high-risk patients with NB, wherein the 3-year event-free survival (EFS) was 61.6% in the tandem transplant group and 48.4% in the single transplant group (*P* = 0.006) [19]. For high-risk patients with NB displaying distant metastases, the high-intensity multimodal treatment methods, such as tandem autologous transplantation and new targeted or immune treatment measures, are recommended for the improvement of their poor prognosis.

When the different metastasis patterns of NB were compared, we found that bone metastasis (42.6%) and liver metastasis (10.9%) were the most common single-organ metastasis, and the combination of bone and liver metastasis in multi-organ metastasis reached 9.5%. On exploring the risk factors of bone and liver metastases, we found that patients without lung metastasis had a lower risk of bone or liver metastasis. Furthermore, although only lung metastasis (1.3%) is uncommon, lung metastases were accelerated when tumours metastasised to other sites (6.6%). Therefore, it was speculated that once the tumour metastasises to one distant tissue, it could accelerate the metastasis in other tissues, which needs to be considered in a clinical setting. There are many conjectures about the mechanism of NB with distant metastasis: (1) NB originates from the sympathetic nervous system; hence, it might distribute along the sympathetic nervous system; (2) NB is an immune cold tumour with multiple immune escape mechanisms, such as Myc and N-Myc downregulating MHC-I to circumvent T cell-mediated cytotoxicity [20] or tumour-associated macrophages and myeloid suppressor cells that drive immunosuppression in NB [21]; (3) genetic variation and epigenetic changes: 1p and 11q deletion, 17q gain, MYCN amplification and the transcriptome up-regulation of N-Myc and down-regulation of EZH2 are closely related to NB metastasis [22–24]; (4) noncoding RNAs are involved in metastasis. For example, lncNB1 promotes the progression of NB by interacting with the ribosomal protein RPL35 [25]. Among them, gene mutation and metastasis of NB have received extensive attention. MYCN amplification was observed in approximately 20 to 25% of NB cases [26]. Moreover, the levels of MYCN correlated with metastatic behaviour. Notably, MYCN contributes to all facets of metastasis: adhesion, motility, invasion and degradation of surrounding matrices [27, 28]. Furthermore, ALK abnormalities were observed in approximately 10% of all sporadic NB [29]. The overexpression of ALK mutants or fusions results in increased migration and invasiveness in NB cells via the upregulation of the MAPK pathway targeting ETV5 [30]. Therefore, it is crucial to elucidate the biological properties of NB and actively explore and develop new and more effective therapies.

Next, we explored the risk factors of patients with bone metastases and liver metastases, respectively, and found that the adrenal primary site was a risk factor for liver and bone metastasis, which could be attributed to the different biological characteristics of NB at different origins, such as ALK mutation levels and MYCN amplification situation [12, 31]. Furthermore, the presence of intra-tumour heterogeneity and different evolutionary trajectories with discrete genotypes in different locations of the same tumour was also observed. Moreover, bone metastasis was more likely to be detected in patients with age > 1 year and was less likely to develop liver metastasis, which is consistent with previous studies [32]. Previous studies have demonstrated age-related changes in MYCN, TERT, PTPRD and Ras pathway alterations, suggesting that the sympathetic nervous system, the tissue of origin of NB, is susceptible to different oncogenic lesions at different times during development [33, 34]. When screening the risk factors, we found that children with NB undergoing surgery had a reduced risk of liver metastasis but had no effect on bone metastasis. Moreover, chemotherapy and radiation therapy were also observed to increase the risk of bone metastasis in NB. Mullassery et al. reported that radical surgery can improve the prognosis of metastatic NB [35]. However, some studies did not observe a survival benefit of surgical resection in patients with high-risk NB [36, 37]. Nonetheless, the reason and mechanism of the unsatisfactory therapeutic effect of chemotherapy and radiation therapy on patients with NB exhibiting bone metastasis require further exploration.

This study acknowledges its limitations. First, missing data in the sample inevitably introduces selection bias. Second, there is a lack of information on chemotherapy regimens, tumour recurrence, immunotherapy, targeted therapy, stem cell transplantation, INSS stage, monthly age and MYCN expansion. Third, owing to the difficulty in obtaining the data on other metastatic sites, only the information available on the liver, lung, bone and brain was used in this study. Finally, this is a retrospective study that requires further prospective, multicentre and large-scale studies to validate our findings.

## Conclusion

The population-based analysis of 1224 patients with NB revealed the incidence of metastasis to be 48.9% of all NB cases. Moreover, metastasis was an independent risk factor for OS and CSS. Bone metastasis and liver metastasis were the common distant metastasis patterns of NB, with age > 1 year, grades III–IV and tumour size 5–10 cm increasing the risk of bone metastasis in NB. Additionally, surgical treatment reduced the risk of liver metastasis in NB. For high-risk NB with distant metastasis, a combination of tandem autologous stem cell transplantation, immunotherapy and targeted therapy is recommended to improve its poor prognosis.

## Data Availability

The datasets used and/or analysed during the current study are available from the corresponding author upon reasonable request.
